# A Type III restriction–modification system in *Mycoplasma mycoides* subsp. *capri*

**DOI:** 10.1098/rsob.120115

**Published:** 2012-10

**Authors:** Mikkel A. Algire, Michael G. Montague, Sanjay Vashee, Carole Lartigue, Chuck Merryman

**Affiliations:** The J. Craig Venter Institute, 9704 Medical Center Drive, Rockville, MD 20850, USA

**Keywords:** methyltransferase, mycoplasma, phase variation, Type III

## Abstract

The sequenced genome of *Mycoplasma mycoides* subsp. *capri* revealed the presence of a Type III restriction–modification system (MmyCI). The methyltransferase (modification) subunit of MmyCI (M.MmyCI) was shown to recognize the sequence 5′-TGAG-3′ and methylate the adenine. The coding region of the methyltransferase gene contains 12 consecutive AG dinucleotide repeats that result in a translational termination at a TAA codon immediately beyond the repeat region. This strain does not have MmyCI activity. A clone was found with 10 AG repeats such that the gene is in frame, and this strain has MmyCI activity, suggesting that the expression of the MmyCI methyltransferase may be phase variable.

## 2. Introduction

Type III restriction enzymes are complexes composed of a methyltransferase subunit (Mod) and a restriction endonuclease subunit (Res) encoded by *mod* and *res* genes, respectively [1]. Using S-adenosyl-l-methionine (SAM) as a methyl donor, the methyltransferase modifies the recognition site independently or complexed with the endonuclease subunit. Restriction activity requires both the Mod and Res subunits and is adenosine-5′-triphosphate (ATP)-dependent. It has been reported that two copies of the recognition site in inverse orientation and on the same DNA molecule are required for cleavage [2].

The genus *Mycoplasma* are wall-less bacteria and are important host-adapted pathogens in a variety of species [3]. Short sequence repeats (SSRs) have been found in the genomes of *Mycoplasmas* and other prokaryotes. These SSRs vary in length and composition, and are often found within the open reading frames of proteins, sometimes altering the reading frame. In such cases, SSRs are indicative of phase variable gene expression [4–10]. Phase variation is the heritable, high-frequency and reversible on/off switching of gene expression. It is common in *Mycoplasma*, and can be mediated by several alternative mechanisms [8,10–12]. During DNA replication, SSRs gain or lose repeat units by slipped-strand mispairing, which changes gene expression by introducing or removing frameshift mutations [8,13]. Host-adapted bacterial pathogens frequently use phase variation to generate diversity in surface structures, such as capsules, lipopolysaccharides and flagella [10,14]. Restriction–modification (R-M) systems are cytoplasmic gene products that can also be modulated by phase variation. The Type I R-M system from *Mycoplasma pulmonis* has been shown to undergo phase variation [15,16]. Numerous Type III R-M systems in host-adapted pathogens, including *Mycoplasma* species, are potentially phase-variable based on sequence analysis [5,17]. It is thought the standard function of R-M systems in the cell is to act as a barrier to the expression of foreign DNA. Phase-variable Type III R-M systems have been suggested to allow temporary removal of this barrier, allowing potentially beneficial DNA to be acquired from the environment [18,19]. It has also been proposed that phase-variable Type III systems can affect global gene regulation through methylation [20].

Here, we identify a Type III R-M system (MmyCI) in *Mycoplasma mycoides* subsp. *mycoides* large colony, which has recently been renamed *M. mycoides* subsp. *capri* [21]. We heterologously express, purify and *in vitro* characterize the methyltransferase subunit, M.MmyCI. MmyCI recognizes and methylates the adenine residue in the four-base sequence 5′-TGAG-3′.

## 3. Results

### 3.1. Sequence analysis of the MmyCI restriction–modification system

**Figure 1.**
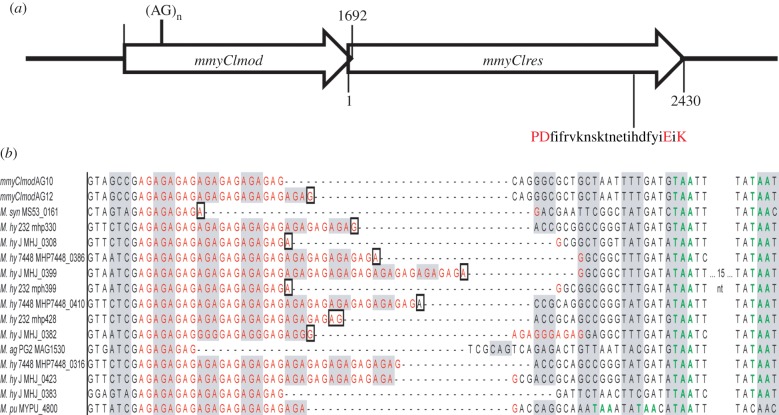
The MmyCI restriction–modification system of *M. mycoides* subsp. *capri*. (*a*) The *mmyCImod* gene is located upstream of *mmyCIres*. The two genes overlap at the 3′ end of *mod*. The AG repeats begin at nucleotide 320 in the *mod* gene. The predicted amino acid sequence of the active site of Res is indicated. (*b*) A zoomed-in view of the sequence alignments using ClustalX of *mod* genes from several *Mycoplasmas* shows a conserved AG repeat of various lengths (red). The alternate shading of grey and white shows the reading frame in which a full-length protein is predicted to be produced. The boxed residues indicate a change in the reading frame of the gene. The conserved TAA stop codons are coloured green. The *M. pulmonis* gene contains one of the conserved stop codons, but has two stop codons upstream that can act to truncate translation. The organism abbreviations are *M. syn* (*M. synoviae* 53), *M. hy* 232 (*M. hyopneumoniae* 232), *M. hy* J (*M. hyopneumoniae* J), *M. hy* 7448 (*M. hyopneumoniae* 7448), *M. ag* PG2 (*M. agalactiae* PG2), *M. pu* (*M. pulmonis* UAB CTIP).

Analysis of the *M. mycoides* subsp. *capri* GM12 genome sequence (Genbank accession no. CP001621) revealed an operon of two genes with homology to the *mod* and *res* genes of Type III R-M systems [22]. The *mmyCImod* gene precedes the *mmyCIres* gene. The two genes overlap at the 3′ end of *mod* by 14 nucleotides. The predicted M.MmyCI protein has motifs characteristic of m6A or m4C methyltransferases [23]. The R.MmyCI protein has motifs characteristic of a DNA helicase [24] and endonuclease [25] (figure 1). Several putative Type III restriction systems have been identified in other *Mycoplasma* species, based on sequence homology [5,22,26], although evidence for activity of the Type III system is only seen for *Mycoplasma pulmonis* strain CT [27]. A characteristic of most of these systems in *Mycoplasma* is the presence of short nucleotide repeats found in the coding region of the *mod* gene (figure 1). These short repeats have been shown to allow phase variation of the genes in several organisms [10]. The originally sequenced *mmyCImod* gene found in *M. mycoides* contains a run of 12 AG dinucleotide repeats within the coding region of the gene (*mmyCImod*-AG12). Variation in the length of the repeat can alter the reading frame of the *mmyCImod*-AG12 gene. Translation of the *mmyCImod*-AG12 gene will result in a truncated protein as a TAA stop codon is encountered immediately following the repeat region. In the sequences examined, there are two conserved TAA stop codons in the –1 and –2 reading frames such that additions or deletions of repeat units that alter the reading frame result in truncated proteins. Subsequently, a new *M. mycoides* clone that had a reduction in the number of these AG repeats in the *mmyCImod* gene was found. The gene from this clone contains 10 AG repeats (*mmyCImod*-AG10). This clone was the progeny of the 12 AG containing *M. mycoides* clone. The removal of two AG repeats from this region changes the reading frame and allows the entire methyltransferase protein to be translated.

*Mycoplasma mycoides* subsp. *capri* genome has been cloned in yeast and was the basis for the first synthetic cell [28,29]. In these studies, this technology was used to generate a *M. mycoides* cell with the *mmyCIres* gene seamlessly deleted in order to remove the endonuclease activity in the organism. The *mmyCIres* gene is not essential for viability and shows no obvious phenotypic differences (such as growth rate and colony morphology). A similar observation was made when both the *mod* and *res* genes were deleted.

To further characterize this R-M system, we purified both the products encoded by the *mmyCImod* (M.MmyCI) and *mmyCIres* (R.MmyCI) genes after expression in *Escherichia coli* (§5). To express the proteins, we constructed a *mmyCImod* gene that would encode the protein product predicted to be produced from the *mmyCImod*-AG10 gene (without the ‘off’ frameshift). The *mmyCImod*-AG10 gene we constructed replaced the UGA codons used to encode tryptophan in *Mycoplasmas* with TGG codons. We also removed the AG repeats in the DNA sequence, but retained the proper reading frame of the protein sequence in order to avoid the on/off switching of the gene while being propagated in *E. coli*.

### 3.2. Determination of the methylation site of MmyCI

We chose to follow the methyltransferase activity of MmyCI as a means to identify the recognition site of the enzyme. Type III Mod subunits can function independently and modify a single recognition site [30], though we found the methyltransferase activity of Mod subunit (M.MmyCI) was greatly enhanced by the presence of the Res subunit (R.MmyCI) to make the entire complex (MmyCI). We examined the ability of the MmyCI complex to methylate DNA with [H^3^-methyl]-S-adenosyl-l-methionine (H^3^-SAM) using a plasmid (pRS426-pMyco1) as a potential substrate. In order to avoid DNA cleavage, the methylation reactions were performed without ATP, which is essential for the endonuclease activity of Type III restriction enzymes. The pRS426-pMyco1 plasmid was methylated by MmyCI (data not shown). Further experiments were necessary to clearly identify the recognition site of MmyCI. To this end, pRS426-pMyco1 was subdivided by using PCR to generate nine non-overlapping (approximately 1 kb) fragments that covered the majority of the pRS426-pMyco1 sequence. These subfragments were used as the substrates in separate methylation reactions with MmyCI and H^3^-SAM. All of the DNA subsections were methylated by the enzyme to various degrees, indicating that the MmyCI recognition site was present in each of the nine subsections. From this result, we reasoned that the recognition site is common, and therefore probably contained four bases. Using computer analysis, 107 four-base DNA candidate sequences were identified by comparing all of the common four-base sequences in the nine DNA subsections. A likely recognition site of 5′-CTCA-3′ (or the compliment 5′-TGAG-3′) was identified based on the agreement between the relative levels of H^3^-SAM incorporation in the various DNA sections and the number of CTCA/TGAG sites they contain.

**Figure 2.**
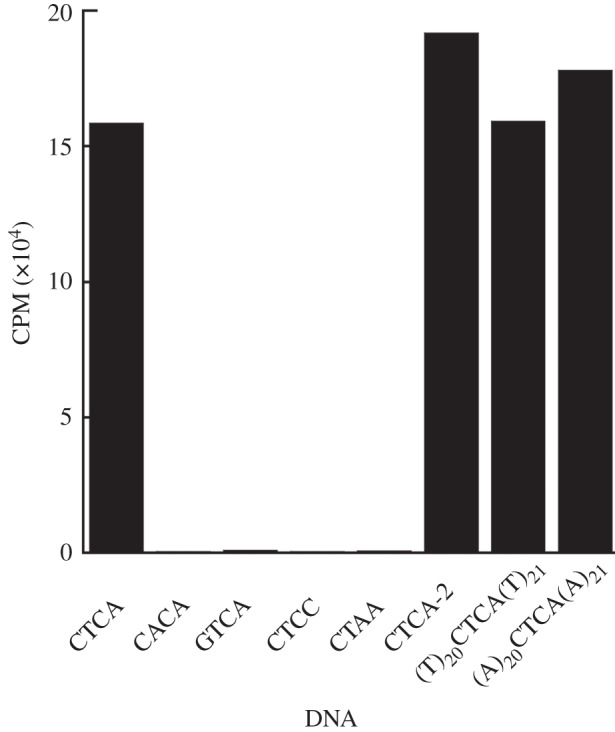
Identification of MmyCI recognition sequence. Scintillation counts of 45 bp DNA substrates following incubation with MmyCI and clean-up indicate that the MmyCI recognition site is TGAG/CTCA (representative values shown).

To investigate this putative site (CTCA/TGAG) directly, we used 45 bp dsDNA oligos that contained the site or similar sites that differed by a single nucleotide flanked by DNA sequence that does not contain the CTCA/TGAG site (§5). The sequence of the top strand of the dsDNA tested was 5′-catgttagcttccgatcagg**CTCA**ggcatcatcatttagtgcgta-3′. Other dsDNAs had the CTCA sequence changed to CACA, GTCA, CTCC, CTAA. Only the CTCA/TGAG containing DNA was substantially methylated (figure 2).

To examine the effect of the flanking sequence on the recognition of CTCA/TGAG site, we performed methylation experiments with 45 bp dsDNAs with changes to the sequence flanking the CTCA site. First, we changed the sequence immediately flanking the CTCA site (underlined residues) 5′-catgttagcttccgatgtca**CTCA**acgttcatcatttagtgcgta-3′. Second, we used dsDNAs with only A residues or T residues surrounding the CTCA site, 5′-(a)_20_**CTCA**(a)_21_-3′ and 5′-(t)_20_**CTCA**(t)_21_-3′. In each instance, the dsDNA was methylated to a similar extent as the original CTCA containing DNA, demonstrating the recognition site for MmyCI is the four-base sequence CTCA/TGAG (figure 2).

**Figure 3.**
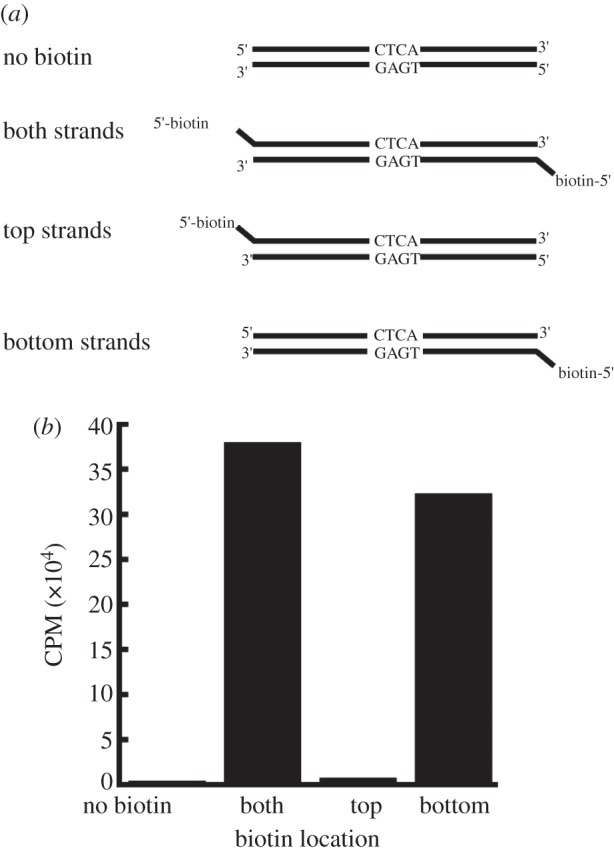
Identification of methylated residue by MmyCI. (*a*) Unlabelled and biotin-labelled oligonucleotides were annealed to generate DNA substrates. (*b*) Scintillation counts of substrates following methylation, competition with biotin-free oligos and clean-up (representative values shown), indicating the adenine residue of 5′-TGAG-3′ is methylated.

MmyCI has the characteristic motifs of an adenine or cytosine methyltransferase. To determine which strand and residue is methylated by MmyCl, the 5′ ends of the 45 bp CTCA/TGAG dsDNA were labelled with biotin in one strand, both or neither. Following methylation with H^3^-SAM, 60-fold excess unbiotinylated oligo was added to the sample and used to compete with the signal of the unbiotinylated strand, allowing the level of H^3^ incorporation of the biotin labelled strands to be measured after purification with Streptavidin agarose resin (see §5 for details). The double biotin-labelled control showed considerable modification while the unlabelled control sample had a low signal, as expected (figure 3). The bottom strand sample signal is comparable to the double biotin-labelled sample, indicating that the bottom strand (TGAG) was methylated. These results and the presence of characteristic m6A motifs in the protein demonstrate that MmyCI recognizes the sequence 5′-TGAG-3′ and methylates the adenine residue.

### 3.3. Differential expression and activity of *mmyCImod*-AG10 and -AG12 clones

The sequences of the *mmyCImod*-AG12 and *mmyCImod*-AG10 genes predict that there should be differential expression of the methyltransferase gene in the AG10 and AG12 clones. This differential expression suggest that the gene could be phase variable. In order to demonstrate this, we examined the presence of MmyCI *in vitro*.

**Figure 4.**
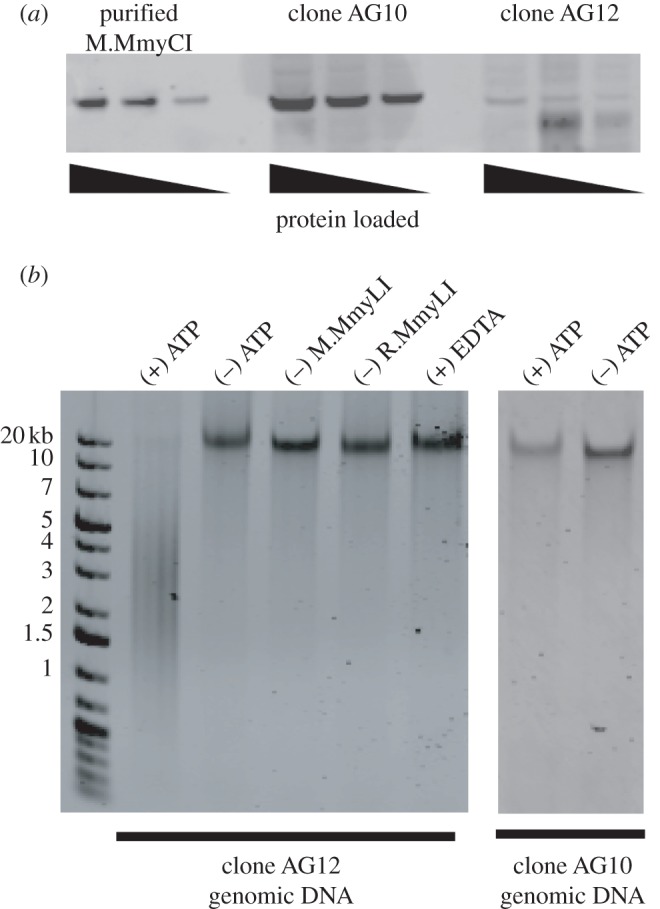
Differential MmyCI expression between clones. (*a*) Western blot analysis of *M. mycoides* lysates. Whole cell lysates of clone AG10 and AG12 were run on a SDS-PAGE gel and transferred to a PVDF membrane for Western blot analysis (50, 25 and 12.5 µg of total protein). Purified M.MmyCI was run as a marker (16, 8 and 2.6 ng). The MmyCI methyltransferase is present in clone AG10 and may be present at a substantially lower level in a population of clone AG12. (*b*) DNA cleavage by MmyCI. Purified *M. mycoides* DNA was used as a substrate for the restriction activity of MmyCI. The complete reaction contained 1 µg of DNA, 1 mM ATP, 1.6 µM MmyCI and 1X NEB buffer four supplemented with 1X BSA. Cleavage activity is not observed in the absence of ATP, M.MmyCI or R.MmyCI, nor in the presence of 20 mM EDTA.

The presence of the MmyCI methyltransferase subunit in the *M. mycoides* clones was determined by Western blot analysis. Lysates were prepared from *mmyCImod*-AG12 and *mmyCImod*-AG10 containing clones (clone AG12 and clone AG10, respectively), and probed with polyclonal antibodies raised against the purified M.MmyCI protein (figure 4). The methyltransferase is produced in clone AG10 cells as predicted by the gene sequence. Clone AG12 shows a faint band at the position where M.MmyCI is expected, suggesting a low level of M.MmyCI in the lysate. This may be due to a small minority of clone AG12 cells that have stochastically altered the number of AG repeats and switched the *mmyCImod* gene ‘on’ or to coincidental background fluorescence.

Purification of MmyCI allowed us to examine the methyltransferase activity of the complex *in vivo*. Cells with active *mmyCImod* genes are expected to have DNA that is protected from MmyCI endonuclease activity, whereas the clones with inactive genes will be susceptible to cleavage. Purified MmyCI was incubated with genomic DNA isolated from the two *M. mycoides* clones (figure 4*b*). DNA from clone AG12 (frameshifted) was degraded by MmyCI. The DNA from the non-frameshift clone (clone AG10) was not completely digested, indicating that *mmyCImod* gene encodes a functional methyltransferase.

Genomic DNA from clone AG12 was used as a substrate to further examine the DNA cleavage activity of MmyCI. Removal of the Mod subunit (M.MmyCI) or the Res subunit (R.MmyCI) abolished the DNA cleavage activity, as expected for a Type III restriction enzyme that requires the presence of both subunits for DNA cleavage activity. The reaction was dependent on the presence of ATP. Also, the complete reaction could be inhibited by the addition of 20 µM EDTA (figure 4*b*).

## 4. Discussion

We have identified a functional Type III R-M system in *M. mycoides* subsp. *capri*, designated MmyCI. MmyCI recognizes and methylates the adenine in the sequence 5′-TGAG-3′. This is a new recognition sequence for a Type III enzyme and this sequence has not been reported for any other R-M system [22].

We have isolated two *M. mycoides* clones that have AG dinucleotide repeats within the *mod* gene. The number of repeats in the gene alters the reading frame, indicating that the *mod* gene could be subject to phase variability by the loss or addition of AG repeat units, although this needs to be demonstrated more thoroughly. It is likely that the putative phase variability of the gene is mediated by slipped-strand mispairing of the dinucleotide repeat region during DNA replication events, similar to the *vmc* genes in *M. capricolum* subsp. *capricolum* [7]. While slipped-strand mispairing appears to be a likely mechanism for changing the ‘on/off’ state of the *mod* gene, the signal or inducer of this change is not known.

Several other *mod* genes from *Mycoplasmas* have been found that also contain various numbers of AG dinucleotide repeats within the coding region. Several of these *Mycoplasma* genes also contain frameshift mutations at the site of their AG repeats, just as the AG12 gene does, suggesting that these genes probably also undergo phase variation. The high degree of homology with M.MmyCI also suggests that these genes encode for functional methyltransferases.

It is thought that bacteria use the phase variability of gene expression to evade the host immune system. The majority of phase-variable gene products from bacteria are located on the surface and are often associated with virulence [10,14]. The biological significance of phase-variable R-M systems is unknown, although a Type III methyltransferase from *Haemophilus influenzae* has been shown to coordinate expression of multiple genes [31]. Also, adenine methylation has been demonstrated to affect virulence of *Salmonella typhimurium* [32]. It may be possible that methylation by MmyCI can effect global expression in *M. mycoides,* although the presence of an active Res subunit implies additional functions.

Complex interactions within the organism and between the pathogen and host make it difficult to determine the exact role that possible phase variation of an R-M system plays in the biology of *M. mycoides* and in bacterial pathogens in general. We hope the recent development of synthetic genomic techniques that allow the use of powerful yeast genetic methods for *M. mycoides* subsp. *capri* may be applied to elucidate the biological implications of combinatorial gene changes and differential methylation in *M. mycoides* [28,29].

## 5. Material and methods

### 5.1. Expression and purification of MmyCI

#### 5.1.1. M.MmyCI

The coding sequence of the methyltransferase of MmyCI (M.MmyCI) identified in *M. mycoides* subsp. *capri* was codon-optimized for expression, which included replacing the TGA codons with TGG and removing the frameshift. The sequence was constructed using a one-step isothermal DNA assembly method [33]. It was cloned into pTYB1 (New England Biolabs; NEB) and transformed into BL21(DE3) codon plus cells (Stratagene). Transformants were grown in 250 ml of ZYM-505 medium [34] containing 100 µg ml^−1^ carbenicillin and 34 µg ml^−1^ chloramphenicol at 37°C with vigorous shaking (315 r.p.m.). After approximately 4 h, the cultures were transferred to 16°C and expression was induced by the addition of 0.3 mM IPTG. The cells were pelleted after overnight incubation, suspended in 50 ml Intein lysis buffer (25 mM HEPES-NaOH pH 7.2, 500 mM NaCl, 1 mM EDTA, 10% glycerol) and complete protease inhibitor cocktail (Roche), then lysed by two passages through a high-pressure homogenizer. The lysate was clarified by centrifugation (20 000 *g*, 20 min, 4°C), and M.MmyCI was purified on a 1.5 ml column of chitin beads, according to the manufacturer's directions (NEB). Fractions containing the methyltransferase were pooled and the elution buffer was exchanged for enzyme buffer (50 mM HEPES-NaOH pH 7.2, 100 mM NaCl, 0.1 mM EDTA, 10% glycerol) using an Amicon Ultra Centrifugal Filter Unit (Millipore).

#### 5.1.2. R.MmyCI

The restriction endonuclease subunit (R.MmyCI) coding sequence was amplified from genomic DNA in three overlapping pieces using primers that replace TGA codons with TGG codons. Isothermal DNA assembly was used to assemble the gene into pTYB1. Expression and purification of the R.MmyCI protein was performed as above for M.MmyCI.

### 5.2. Methylation assays

Standard methylation reactions (100 µl) contained 1 µM dsDNA oligo or 1 µg PCR product, 120 nM H^3^-SAM, 500 nM MmyCI and 1X NEB buffer 4. The reactions were incubated at 37°C for 2 h and terminated with 100 µl of phenol. Unincorporated H^3^-SAM was removed by passing 70 µl of the aqueous phase through P-30 Micro Bio spin columns (BioRad). The [H^3^-methyl]-labelled DNA was quantified by scintillation counting.

The sequence of the top strand oligo used to verify the recognition site is 5′-catgttagcttccgatcagg**CTCA**ggcatcatcatttagtgcgta-3′. CTCA was changed to CACA, GTCA, CTCC or CTAA in the control DNAs. The sequence of the top strand oligos used to examine the effect of the flanking sequence on the recognition site were 5′-catgttagcttccgatgtca**CTCA**acgttcatcatttagtgcgta-3′ (underlined nucleotides different from above), 5′-(a)_20_**CTCA**(a)_21_-3′ and 5′-(t)_20_**CTCA**(t)_21_-3′.

Methylation reactions with biotin labelled DNA were performed using 500 nM duplex DNA. The reactions were stopped and the H^3^-SAM was removed as described above. Excess biotin-free oligo was added to a final concentration of 30 µM, the samples were heated at 95°C for 5 min, then placed on the bench top and allowed to cool. Approximately, 50 µl of Streptavidin agarose resin (Thermo Scientific) was used to isolate the biotin-containing DNA. Unbound DNA was removed by washing the resin with 4 ml of H_2_O. After washing, the resin was resuspended in 100 µl H_2_O and co-purifying radiolabel was measured.

### 5.3. Sequence analysis

Type III R-M systems in *Mycoplasma* species were identified from REBASE [22]. DNA sequences were acquired from Genbank, and initially aligned with ClustalX v. 2.0 [35]. The alignment was then refined manually in the vicinity of the AG repeats. Sequences that did not contain the AG repeat region were then removed from the analysis; these genes were also noted as having relatively divergent sequences. The sequences that remained were the *mod* genes corresponding to locus tag MAG1530 from *Mycoplasma agalactiae* PG2 (NC_009497.1); locus tags mph330, mph399 and mhp428 from *Mycoplasma hyopneumoniae* 232 (NC_006360.1); MHP7448_0316, MHP7448_0386 and MHP7448_0410 from *M. hyopneumoniae* 7448 (NC_007332.1); MHJ_0308, MHJ_0382, MHJ_0383, MHJ_0399 and MHJ_0423 from *M. hyopneumoniae* J (NC_007295.1); MYPU_4800 from *Mycoplasma pulmonis* UAB CTIP (NC_002771.1); and MS53_0161 from *Mycoplasma synoviae* 53 (NC_007294.1). The genes *mmyCImod*-AG10 and *mmyCImod*-AG12 from *M. mycoides* were also included in the alignment.

### 5.4. Western blot analysis

Purified M.MmyCI (16, 8 and 2.6 ng) or a lysate of *M. mycoides* cells (50, 25 and 12.5 µg of total protein) were electrophoresed on 4 to 12 per cent Bis-Tris polyacrylamide gels (Invitrogen) and transferred to an Immobilon-FL PVDF membrane. The membrane was incubated in TBST (50 mM Tris pH 7.5, 150 mM NaCl, 0.05% Tween-80) containing 5 per cent BSA for 2 h at room temperature. The membrane was then incubated with mouse polyclonal antibodies raised against purified M.MmyCI (Precision Antibody, Columbia, MD, USA) for 1 h, washed three times with TBST and probed with Alexa Fluor 555 donkey antimouse IgG (Invitrogen) for 1 h. After three washes with TBST, the protein was visualized with a Typhoon 9410 imager (GE).

### 5.5. DNA cleavage assays

DNA cleavage reactions typically contained 300 ng to 1 µg of substrate DNA, 0.5 mM ATP, 500 nM MmyCI complex (500 nM M.MmyCI and 500 nM R.MmyCI) and 1X NEB buffer 4. The reactions were incubated at 37°C. In order to quench the reactions, 25–50 µl was added to an equal volume of buffer-saturated phenol containing 0.1 M EDTA. After quenching, the aqueous phase was removed and purified with the QIAquick PCR purification kit (Qiagen). The products were separated with a 0.8 or 1.2 per cent e-Gel (Invitrogen).

## 6. Acknowledgements

We thank Rich Roberts for initial identification of the Type III operon. We also thank the Synthetic Biology team for critical reading of the manuscript. This work was supported by Synthetic Genomics, Inc. M.A.A., M.G.M. and C.M. wrote the main manuscript text. M.A.A. supervised the project. All authors performed experiments and analysed data. All authors reviewed the manuscript.
